# Improving mental health literacy in secondary school educational professionals in the netherlands: adaptation of an existing intervention using Intervention Mapping Adapt

**DOI:** 10.1186/s12889-025-23835-5

**Published:** 2025-09-02

**Authors:** Janne M. Tullius, Bas Geboers, Jacomijn Hofstra, Lies Korevaar, Sijmen A. Reijneveld, Andrea F. de Winter

**Affiliations:** 1 Department of Health Sciences, University Medical Center Groningen, University of Groningen, Antonius Deusinglaan 1/FA10, Groningen, 9713 AV The Netherlands; 2 Research and Innovation Centre for Rehabilitation, Hanze University of Applied Sciences, Groningen, The Netherlands

**Keywords:** Adolescent, Health literacy, Help-seeking behavior, Mental health, School teachers, Secondary schools

## Abstract

**Background:**

Advanced interventions to enhance the mental health literacy (MHL) of educational professionals are now available, though their effectiveness varies, likely due to contextual differences. To adapt these interventions for use in other countries, a systematic and tailored approach is required to maintain the logical framework. This study describes the systematic process of selecting and adapting an intervention to improve MHL of Dutch secondary school educational professionals, using Intervention Mapping (IM) Adapt.

**Methods:**

IM Adapt regards six steps: (1) assess needs and organizational capacity; (2) find evidence-based interventions; (3) plan adaptations; (4) make adaptations; (5) plan for implementation; and (6) plan for evaluation.

**Results:**

The outcomes of a needs assessment were used to develop a logic model of the problem and of change, leading to the intervention goal of increasing mental health awareness, knowledge, attitudes, and self-efficacy among end-users. A logic model of the problem and of change visually outlines the relationship between a specific issue, its root causes, and the intended intervention to drive change. The existing evidence-based digital intervention, named LEARN, was identified as the most suitable for addressing these needs. This was done through reviewing the literature and existing intervention databases. Four main content adaptations were made to align with the distinct needs of the Dutch context and the evolving trends within the field of mental health, resulting in LEARN-NL, a digital mental health literacy intervention for Dutch educational professionals. Finally, implementers were identified, and a mixed methods feasibility study was set up.

**Conclusions:**

Our findings show that some content adaptations to LEARN were needed to address the needs and (learning) preferences of educational professionals in the Dutch education context, but that the majority of the existing intervention could be upheld. Existing evidence-based MHL interventions for educational professionals are useful as a basis for the adaptation and transfer to other countries. IM Adapt is a valuable framework for the systematic planning of adaptations to a new context while retaining its essential elements.

**Supplementary Information:**

The online version contains supplementary material available at 10.1186/s12889-025-23835-5.

## Background

Mental health problems among adolescents are a common and widespread public health concern. It is estimated that worldwide 10–25% of all young people between the ages 10 and 18 experience mental health problems [1, 2]. Experiencing mental health problems during adolescence can have a long-lasting detrimental impact on young individuals’ lives with regard to their future health, social participation, and employment opportunities, in particular when left unidentified and untreated [3–5].

Educational professionals in the secondary school environment (e.g., educators, counselors, social workers, care team members) are in a prime position to identify signals of mental health problems and support adolescents in their help-seeking decisions and behaviors [6, 7]. Unfortunately, educational professionals often do not feel well-trained and competent to identify and support adolescents with mental health problems [8–11]. Equipping educational professionals with better mental health knowledge and skills can enable them to play an instrumental role in identifying secondary school students who may be at risk for mental health problems, responding to their needs effectively, and having productive collaborations with other stakeholders (e.g., mental health professionals, parents) when faced with student mental health challenges [7]. It can also stimulate the creation of a classroom environment where students feel safe discussing their mental health [7].

The necessary knowledge and skills are often referred to as mental health literacy (MHL), “the range of cognitive and social skills and capacities that support mental health promotion”, which has gained recent attention in the realms of mental health promotion in the school environment [12, 13]. MHL comprises four domains ‘1) understanding how to obtain and maintain positive mental health, 2) understanding mental disorders and their treatments, 3) decreasing stigma related to mental disorders, and 4) enhancing help-seeking efficacy (knowing when and where to seek help and developing competencies designed to improve one’s mental health care and self-management capabilities)’ [14]. MHL includes the capacity to promote not only one’s own personal mental health but also that of those in their immediate surroundings, such as students. To develop MHL in educational professionals, it is important for them to receive ongoing and targeted MHL training [12, 13, 15].

In the last decade, several interventions have been developed to improve the MHL of educational professionals that effectively improved knowledge, helping behavior and confidence, and reduced stigma [12, 16–18]. The majority of these interventions have been developed and evaluated in North America (e.g., United States, Canada) and Asia (e.g., Japan, Hong Kong), with only a few in Europe (e.g., Germany, Hungary, UK) [12, 16]. The positive outcomes of these interventions present an opportunity for contexts that have no MHL interventions for educational professionals thus far, such as secondary education in the Netherlands. Despite the development and implementation of numerous mental health interventions in the Netherlands, the majority focus on identifying and treating youth already struggling with mental health issues or specific disorders such as depression or anxiety [19]. As a result, there’s a noticeable absence of initiatives focusing on universal primary prevention or enhancing MHL, particularly for adolescents and educational professionals. Adapting and implementing evidence-based interventions aiming at improving MHL of educational professionals may fill this gap.

However, the reuse of evidence-based interventions in another context requires a carefully planned and executed adaptation process, as the intervention should be tailored to the specific context, while preserving the intervention’s essential components [20, 21]. If done well, the adaptation of effective interventions, rather than developing new interventions, has the potential to transfer the previously established program effectiveness to a new context while optimally utilizing resources by leveraging existing evidence [20]. In contrast, if not executed properly, the adaptation of interventions also bears risks such as ineffectiveness or even harm of an intervention in the new setting [21].

The Intervention Mapping (IM) Adapt approach, derived from the widely used Intervention Mapping (IM), entails a stepwise, systematic framework that enables the systematic adaptation of evidence-based interventions [20, 22]. However, IM Adapt is still not often used for intervention adaptation [23, 24], despite its counterpart IM being a gold standard for intervention development [24]. IM Adapt provides concrete guidelines for planners of interventions that identify existing, fitting interventions that can be adapted to their population, setting, and cultural context [25]. IM Adapt therefore offers a sound methodology to systematically adapt existing evidence-based MHL interventions for educational professionals to a context in which none of its kind are currently available, such as the Netherlands as described earlier. Following and reporting the steps of IM Adapt may provide transparency in the design process of MHL interventions for educational professionals and ensure careful adaptation processes while preserving essential components.

In this article, we therefore describe the process of the systematic selection and adaptation of an intervention to improve MHL of educational professionals in secondary education in the Netherlands.

## Methods

### Intervention mapping adapt

In this study, we use the IM Adapt protocol to adapt an existing mental health literacy intervention for Dutch secondary school educational professionals. The IM Adapt protocol is characterized by an iterative process for the adaptation of interventions. It consists of a six-step procedure, in which several different methods are employed, including literature research, expert consultations, and mixed methods (Table 1). This article describes all steps of IM Adapt [20]. However, while the needs assessment (step 1) and the mixed methods evaluation study (step 6) were performed in separate studies, this article summarizes the key results and conclusions from steps 1 and 6 in the following sections to create a more coherent narrative of the intervention adaptation. The complete studies on steps 1 and 6 are reported elsewhere in more detail [11, 26]. All steps inherent to IM Adapt were followed by the first author (JMT) in collaboration with the co-authors (AdW, LK, JH, BG, SAR). An advisory board consisting of eight members in total was formed and repeatedly consulted throughout the steps: Two Dutch secondary school educational professionals, three mental health care professionals and two education advisors. The advisory board members were drawn from the researchers’ existing networks and selected for their expertise and interest in youth mental health, education, or both. Those consultations were held through advisory board meetings or by individual meetings with the first author (JMT). All steps were performed between May 2020 (step 1) and December 2023 (step 6).

The study was performed in accordance with the Helsinki Declaration. The study was deemed exempt from human subjects’ review by the Medical Ethical Committee of the University Medical Center Groningen (no. M20.252893).

**Table 1:** Intervention mapping adapt: goals per step in IM adapt and methods used per step in the current study

IM Adapt step	Goal	Method/tool used in this study
1) Needs assessment	To gather relevant information and data about the target population’s needs, preferences, and characteristics related to the health issue or problem.	Qualitative study: Online focus group discussions (*n* = 21 adolescents; and *n* = 12 educational professionals) [11]
2) Find evidence-based interventions and assess fit	To identify an evidence-based intervention that aligns with the goals and determinants among the priority population.	Literature search (Systematic reviews, reports) Advisory group consultations
3) Plan adaptations based on fit assessments	To determine the fit of the identified evidence-based intervention for the new context and make plans for adaptations.	Advisory group consultations
4) Make adaptations	To modify intervention materials so that they are culturally relevant and appropriate for the target population. To ensure that the intervention is tailored to meet the needs and preferences of the specific target group.	Advisory group consultations End-user consultations
5) Plan for implementation	To develop a plan to implement the program among end-users	Advisory group consultations Implementer consultations
6) Plan for evaluation	To develop a plan to assess whether the intervention achieves its intended outcomes and to make any necessary adjustments based on the evaluation findings.	Mixed-methods study [26]

### Procedure

#### Step 1

Prior to the intervention adaptation, a qualitative study was conducted to explore adolescents’ and educational professionals’ experiences with, and perspectives on, mental health help seeking and their needs regarding MHL interventions. This was done by holding online focus group discussions with both adolescents (*n* = 21; 13–19 years) and educational professionals (*n* = 12) and analyzing the data using directed content analysis. The results of this study informed the further steps towards the systematic adaptation of an educational professional MHL intervention [11].

The research team translated the results of the needs assessment together with findings from the literature into a logic model of the problem. Next, the logic model of the problem was converted to objectives in a logic model of change. Changes in specific desired behaviors of the environmental agents (e.g., educational professionals or ‘end-users’), also called performance objectives, and changes in determinants, also called change objectives, were defined. A logic model of the problem and of change is meant to give a careful description of the problem at hand and to depict the lifestyle, health, environmental and/or behavioral factors that contribute to the problem, and to then translate these into specific change objectives [24].

#### Step 2

The existing literature was searched to identify suitable existing evidence-based interventions that aligned with the goals and objectives defined in the previous step. No systematic review was performed in this step; however, recent, existing systematic reviews of MHL interventions for secondary education professionals were identified, and their results used as a pre-selection of candidate intervention. The research team then reviewed the candidate interventions, appraised their quality and their fit with the previously identified change objectives. An intervention was chosen that (1) was most in line with our performance and change objectives, (2) has previously been adapted to and evaluated in different cultural contexts, and (3) could be extended with an existing adolescent MHL intervention (whole school approach). The identified intervention was discussed within the research team in the light of its relevance and fit in terms of program objectives and goals. After reaching consensus, the intervention was then used for the remainder of the steps. This decision was once more verified with the advisory group in step 3.

#### Step 3

The adaptations to the existing intervention were planned in consultation with the advisory board. During the first advisory board meeting (July 2022), first, the performance and change objectives were presented to the members along with the defined program goals for the intervention. The advisory board was asked to reflect on these in terms of adequacy for the Dutch setting and make suggestions for any changes or additions. Second, the research team presented the main topics, learning objectives, and activities of the identified intervention. The participants were then asked to judge its fit for the Dutch setting and population and identify the elements that they perceived as essential. This was done by an activity where paper cards on which the learning objective and/or activity was printed had to be rated on a grading scale from 1 (not at all important) to 10 (essential). If a card was rated as “not at all important”, the researchers flagged the content as a candidate for omission. It was further discussed what intervention format (e.g., face-to-face meetings, frequent workshops, eLearning) would be most desirable for the target group.

The meeting was audio-recorded. Field notes were taken as well. The results of the meeting led to a selection of adaptations to the intervention. The advisory board meeting lasted for two hours and was held at the research location.

During a follow-up advisory board meeting in November 2022, an overview of the adaptations was presented to the advisory board members. Prior to the meeting, the members received one of the modules (Module 5) to review and comment on which was discussed during the advisory board meeting. The planned and executed adaptations were presented to the board members, and their input was asked on the adaptations made. Then, Module 5 was discussed in more detail, specifically what information was deemed necessary due to the module’s lengthy texts and elaborate theory. The meeting was audio-recorded, and field notes were taken. The advisory board meeting lasted for 1.5 h and was held at the research location.

#### Step 4

First, the intervention materials were translated to the Dutch language. Second, the team reviewed the original intervention, marked where adaptations were needed, added, or edited materials and activities in, and made them visible. Adaptations were also presented to the developers of the original intervention to discuss any inconsistencies or get further information to make adaptation decisions. The adapted intervention was reviewed by researchers and educational professionals who gave feedback for final adaptations.

Finally, the intervention underwent a last proofreading by the research team followed by a pre-testing in which two educational professionals were asked to test parts of the intervention and give feedback on its usability and functionality.

#### Step 5 & 6

An implementation plan was formulated by the research team considering to whom, when, and how the intervention would be delivered to the target groups. An evaluation plan to determine the feasibility and effectiveness of the adapted intervention among end-users was formulated.

## Results

In this section, the results of the six steps of the IM Adapt protocol are presented.

### Step 1: Needs assessment

In a previous study, we performed a needs assessment that revealed a pressing need for improving the MHL competencies of educational professionals in secondary education [11]. The needs assessment explored adolescents’ and educational professionals’ experiences with, and perspectives on, mental health help seeking and their needs regarding MHL interventions. During the needs assessment, three main themes were identified: (1) Adolescents perceive their MHL competencies as too limited, (2) educational professionals have limited MHL competencies to provide mental health support, and (3) mental health promotion in the school environment is limited. It was concluded that improving the MHL competencies of both adolescents and educational professionals has the potential to facilitate adolescents’ mental health help-seeking, and that their needs ought to be considered in the adaptation of a MHL intervention. The complete study and results can be found elsewhere [11].

The research team decided to focus on adapting a MHL intervention for educational professionals in the Netherlands before doing so for the adolescent target group, based on extensive consultations with mental health researchers and experts. This decision was prompted by the pressing lack of sufficient mental health training for educational professionals [11, 19] and the recognition that prioritizing their training is essential to equip them with the skills needed to deliver future mental health literacy interventions for adolescents effectively in the classroom [18].

**Fig. 1:**
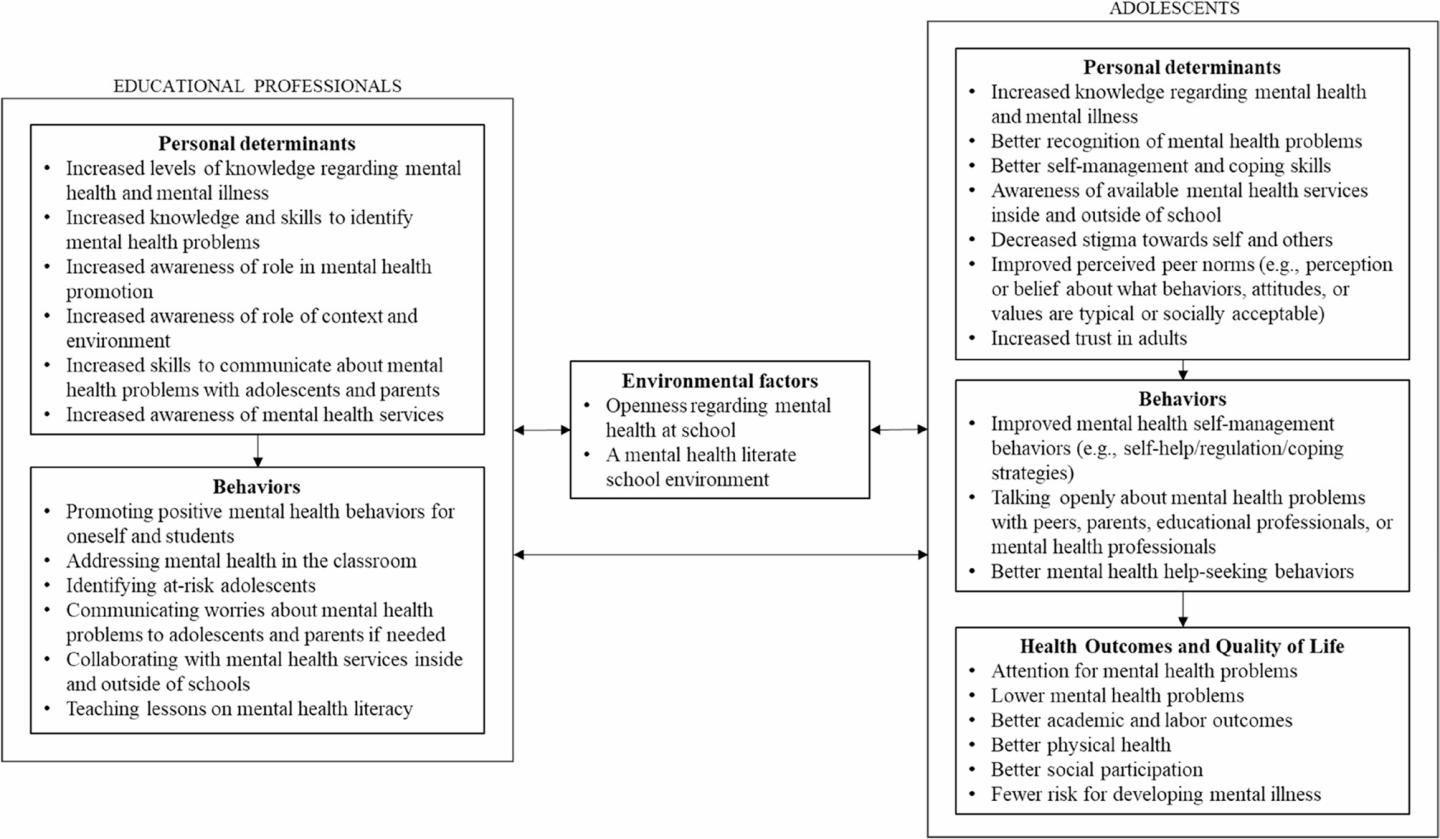
Logic model of change illustrating change objectives of both educational professionals and adolescents

We translated the results of the needs assessment into a logic model of the problem. The intervention goal was determined as the improvement of MHL of educational professionals in terms of awareness, knowledge, attitudes, and self-efficacy. Next, we derived a logic model of change to reach that goal (see Fig. 1).

### Step 2: Identification of suitable evidence-based intervention

The ‘LEARN’ intervention, which is based on a range of Canadian-based school MHL intervention approaches [27], was identified fitting the defined logic model of change and intervention goals (step 1). We identified this intervention by reviewing existing reports and systematic reviews of MHL interventions for educational professionals working in secondary education [12, 28]. A synthesis of these reviews showed that there are six unique evidence-based interventions available that aim to improve educational professionals’ MHL and are of acceptable quality. After reviewing the presented available interventions, the MHL intervention ‘LEARN’ (also known as ‘The Guide (Pre-Service) Professional Development Program’) was selected by the research team because (1) it focuses on the improvement of MHL in educational professionals, (2) it is most in line with the logic model of change, (3) its base materials have previously been adapted to and evaluated in different cultural contexts, (4) it can be extended with an existing, evidence-based adolescent MHL intervention (the Guide) [29, 30], and (5) it holds a digital format to stimulate flexibility, sustainable intervention implementation, and modern learning methods.

‘LEARN’ is a digital MHL resource delivered through a Massive Open Online Course (MOOC) format that comprises seven modules that aim to “enhance MHL of teacher candidates and practicing educators, provide classroom congruent materials that can be used to help address MHL of students, and identify strategies that can be used for maintaining one’s mental health” [31]. LEARN consists of the following seven modules:

- Module 1: Introduction & Background.
- Module 2: Stigma & Mental Health.
- Module 3: Human Brain Development.
- Module 4: Understanding Mental Health, Mental Illness & Related Issues in Young People.
- Module 5: What is Treatment?
- Module 6: Seeking Help & Providing Support.
- Module 7: Caring for Students & Ourselves.

The intervention is derived from the materials of the well-regarded MHL resource ‘Mental Health & High School Curriculum Guide’ (the Guide), created by mentalhealthliteracy.org in collaboration with the University of British Columbia, Faculty of Education, Canada. Wei et al. (2020) reported that the use of LEARN has demonstrated positive outcomes in improving knowledge, improving attitudes (decreasing stigma) and enhancing help-seeking in preservice teachers. Moreover, upon completing the ‘LEARN’ intervention, educational professionals are equipped to effectively implement the materials of an evidence-based adolescent MHL intervention in their own classrooms to enhance students’ mental health literacy [18, 32].

Other candidate interventions employed different delivery methods, including face-to-face single-day training sessions that appear to be effective but are often time-intensive and rarely fit within the daily practices of an educational professional in the school setting [28]. ‘LEARN’ is quite unique in that educational professionals can access the online modules at their convenience, allowing for greater flexibility to meet their educational needs.

### Step 3: Planning of adaptations

Adaptations to the existing intervention were planned in consultation with the advisory board. The advisory board further commented on the content of the original intervention and rated the importance of the contents to identify core elements (e.g., rating paper cards with activities). It was advised to adapt extensive information that might be too theoretical and detailed for educational professionals (e.g., treatments and diagnoses of mental illnesses) or content that might deserve a more prominent role within the intervention (e.g., theory on stress response and burn-out prevention for educational professionals). The advisory board gave preference to upholding the digital format for the intervention due to its flexibility, time efficiency, and effectiveness. Lastly, the extensiveness of the intervention was discussed, ultimately resulting in the recommendation to minimize textual content as much as possible.

During the follow-up advisory board meeting in November 2022, further advice was given for improvement, which consisted mainly of textual adaptations to increase structure and accessibility. A full overview of the planned adaptations derived from the advisory board is listed in Appendix A.

### Step 4: Making adaptations

The original intervention was adapted after review and advice on learning objectives, activities, theoretical text, videos, and supplementary materials. Most of the original content and its online format were retained, but four main adaptations were made, next to several minor adaptations: First, we removed Module 5 on treatments of mental disorders as it was found to be overly detailed and thus not sufficiently tailored to the role and responsibilities of Dutch educational professionals. While educational professionals should be able to recognize signs of mental health problems and disorders, know of appropriate help services to refer students to, and have basic knowledge on the impact of treatments on their students, they are not in a position to diagnose mental disorders or make claims about diagnoses. Information on treatment that was perceived as important by the research team and stakeholders was integrated into other modules.

Second, the module ‘Caring for Ourselves and Our Students’ that discusses the stress response and positive mental health behaviors of both students and educational professionals was placed as Module 2 instead of the original Module 7 to give these topics a more prominent role in the intervention. Recently, the emphasis on the positive mental health perspective, opposed to the ill-mental health perspective, as well as the importance of educational professionals’ wellbeing has largely increased which is reflected in this decision [33, 34].

Third, the subsection on common mental illnesses in childhood of the original intervention was omitted to focus on common mental health illnesses in adolescence. This was done as the future end-users—educational professionals in secondary education—primarily interact with adolescents rather than children.

Finally, in the adapted intervention we removed or adapted text that endorsed mainly the biological perspective of mental disorders or extensively explained neurological processes (Module 4). These adaptations were made to divert from portraying brain functioning as the sole cause of mental disorders [35, 36]. Alternatively, we have directed this module towards the brain development during adolescence and its influence on (learning) behaviors. We further adapted contents regarding issues that are quite context-specific such as norms and laws pertaining to mental health, drug and alcohol use, confidentiality, as well as the intricacies of the Dutch education and mental health care system, including its key players and the existing referral system. Contents were only removed or rigorously adapted after consulting the original developers of LEARN and the advisory board to preserve the potential core elements.

**Fig. 2:**
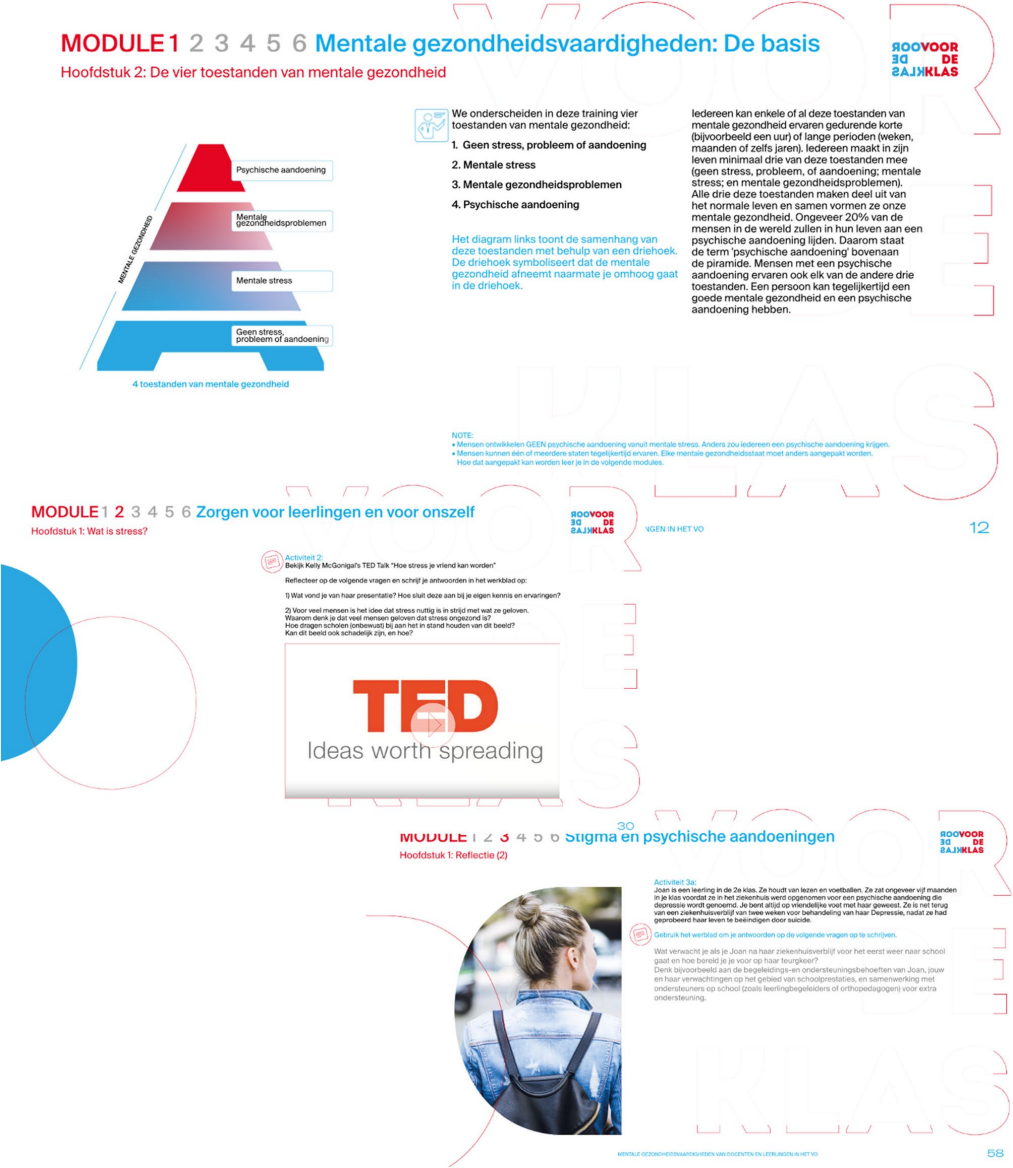
Screenshots of the adapted MHL intervention LEARN-NL for Dutch educational professionals

The materials were then translated into Dutch and converted into a digital, interactive format, including videos, animations, self-evaluations, and additional digital resources. The research team reviewed the materials and made final adjustments, followed by consultations with two educational professionals to review Modules 1 and 3 as proxies for the remaining modules. They provided feedback to ensure clarity and functionality. Overall, the feedback was positive, with minor technical issues addressed, such as activating webpage links. The final adapted intervention in Dutch is called ‘VOOR DE KLAS: Mentale gezondheidsvaardigheden voor docenten in het voortgezet onderwijs (in eng: “In front of the class: Mental health literacy of educators in secondary education” or ‘LEARN-NL’). Screenshots of LEARN-NL are shown in Fig. 2.

### Step 5: Implementation plan

Next, we planned the implementation, addressing two target groups, i.e., secondary schools and institutions that host teacher education programs in the Netherlands. First, secondary schools in the Netherlands will be informed about LEARN-NL through the researchers’ networks, and via newsletters and social media. Schools will be encouraged to have their staff engaged in the intervention. School administrations and school team leaders (e.g., care coordinators, student counselors, orthopedagogues) in secondary education were identified as the main points of contact for the implementation of the intervention at their school. Second, institutions that host teacher education programs in the Netherlands (e.g., universities and universities of applied sciences) will be informed. The goal is to include the intervention in their curricula for teacher education and professionalization.

Program leaders, administrative staff, and curriculum developers and evaluators were identified as the main points of contact for implementation at those institutions. Both schools and teacher training programs will stay in touch with intervention developers for further intervention evaluation. The aim is to have 150 educational professionals complete the intervention in a year. Progress is currently underway in both our pursuit of this objective and the realization of the implementation plan.

### Step 6: Feasibility study

Finally, we made a plan to assess the feasibility of LEARN-NL in the Dutch context, regarding acceptability, implementation, and preliminary effectiveness among users. This mixed-methods study and its results are reported elsewhere [26].

## Discussion

In this article, we described the process of systematic adaptation of an existing intervention to improve the MHL of educational professionals in secondary education in the Netherlands. We found that content adaptations were needed to address the needs and (learning) preferences of educational professionals. Researchers, health promotors and other stakeholders should be aware that an adaptation process is needed to ensure that interventions are aligned to the needs, preferences, and values of end-users in a new context. In this section, we also critically reflected on the value of using IM Adapt as a systematic approach to adapt evidence-based interventions to a new context. We valued the systematic and participatory approach of IM Adapt, although it is time-consuming, as it potentially leads to more effective and suitable interventions.

### A digital mental health literacy intervention for Dutch educational professionals

Our efforts have yielded a MHL intervention for educational professionals that was adapted to the new Dutch context. Most of the original content and its online format were retained, but text and country- and language-specific resources and information were modified. We performed four adaptations to the content of the original intervention to align the content with the specific needs of educational professionals in the Netherlands, as identified through the needs assessment and advisory board consultations. These distinct needs regarded norms and laws specific to the Dutch education and mental health care system, including confidentiality, communication with students and parents, drug and alcohol use, and the existing referral system. These adaptations aimed at enhancing the intervention’s relevance to local end-users and increasing its future uptake. Additionally, some adjustments were made to reflect the current shifts within the field, particularly on how the causes of mental disorders are attributed (i.e., biopsychosocial model rather than biological model) and the growing emphasis on promoting positive mental health [33, 37]. The original digital format by means of an online course was sustained in the adaptation process, allowing for greater flexibility in line with needs of educational professionals [11, 38, 39].

Overall, the adaptations have led to a digital intervention tailored to the Dutch context, called LEARN-NL, that aligns with the needs and motivations of educational professionals, i.e., a pivotal requirement for successful implementation and effectiveness [20, 25]. Despite these changes, we preserved the logic model behind the original intervention and its essential elements to maintain the potential for transferring the previously established program effectiveness. Future assessment of the effectiveness of this adapted intervention for the Dutch setting must shed light on the impact of these adaptations.

### Reflection on Intervention Mapping (IM) Adapt

IM Adapt proved to be useful in shaping the systematic adaptation process of the existing intervention and tailoring it to the needs of the end-user and its new cultural context, while preserving its essential effective elements [20]. The framework of IM Adapt was valuable for two reasons. First, IM Adapt provided clear guidance in the complex process of adapting an existing evidence-based intervention that helped to specify the process required for the further refinement of the existing intervention. Second, IM Adapt further promoted a participatory approach which resulted in frequently consulting stakeholders and experts of different occupational backgrounds, expertise, and thus perspectives. Although this necessitates a significant investment of time and organizational resources, this has enhanced and broadened our understanding of the specific mental health education needs of Dutch educational and mental health professionals, guiding the adaptation of materials to address these distinct priorities in our cultural context. Such a participatory and systematic approach is likely to lead to improved uptake, implementation, and effectiveness of the intervention within the new setting [20, 22].

Several conditions should be taken into account when aligning using the IM Adapt Framework. First, utilizing such a framework is a time-consuming process that demands a substantial allocation of resources, the collaboration of stakeholders, and effective communication with the original developers. Furthermore, while we advocate for a participatory approach that involves stakeholders and experts in the adaptation process, this approach also necessitates a significant investment of time and organizational resources. While stakeholders and experts often provide invaluable insights, some suggestions may not always be feasible or may extend beyond the original scope of the intervention. In such cases, the research team should make final decisions, to maintain the fidelity of the original intervention and ensure to retain its essential elements. It remains to be determined whether this approach has affected the intervention’s effectiveness.

### Strengths and limitations

This study holds a number of strengths. First, a participatory approach was employed, meaning that different stakeholders including educational and mental health professionals were consulted frequently in the adaptation process. Doing so ensured the tailoring and applicability of the contents and methods to the end-user and amplified the quality of the intervention. Second, due to the use of a systematic approach in adapting an existing intervention and careful considerations of its adaptations, we have potentially succeeded in upholding the existing intervention’s fidelity and therefore probably its previously found effectiveness. Third, to the best of our knowledge, this is the first study that has adapted an existing MHL intervention targeting educational professionals in a systematic manner using Intervention Mapping Adapt. Our work therefore highlights and encourages the use of a systematic adaptation process of evidence-based interventions when transferring them to a new country or population. Lastly, we have developed the first mental health literacy intervention targeting educational professionals in the Netherlands.

Worth noting are also the limitations of this study. First, despite our best efforts to involve the advisory board extensively in the decision-making process during adaptations, some members did not have the opportunity to review all materials of the intervention due to time constraints and the comprehensive nature of the materials. Consequently, only a limited number of detailed perspectives from certain members could be considered during the adaptation process, potentially resulting in small-scale adaptations that not all members were aware of and could comment on. Second, although educational professionals were frequently consulted during the adaptation of LEARN, there was minimal input from secondary school students regarding what they considered important for their educators to learn, beyond the initial needs assessment. As a result, while LEARN may be well-tailored to meet the needs of educational professionals, it remains unclear to what extent it also addresses the skills and knowledge that students value in their educators.

### Implications

Our work has several implications. First, our promising findings provide a major opportunity to adapt evidence-based MHL interventions to different contexts. This can be achieved by adapting and transferring existing ones to new contexts and populations. This report of an adaptation process may inform future adaptations or advancements of MHL interventions for educational professionals. It remains to be noted however that adaptations to an existing evidence-based intervention should be carefully planned and executed to fit the specific context while upholding its fidelity. In sum, based on our experiences using IM Adapt, we advise developers of evidence-based interventions to explore the possibility of systematically adapting and re-using existing interventions to a new context or population.

Second, future efforts will focus on broader implementation of the intervention based on the established feasibility of LEARN-NL assessed in IM Adapt. As the intervention demonstrated feasibility, it holds the promise of extensive implementation within Dutch schools. Such an endeavor could significantly enhance the mental health literacy of the overall school environment, instill greater confidence in the capabilities of educational professionals, and ultimately foster improved self-management skills and help-seeking efficacy among students.

Finally, our successful use of IM Adapt to systematically adapt an intervention to a new cultural context using IM Adapt may serve as an example for future intervention developments or adaptations. Intervention developers/adapters in other countries and settings are encouraged to systematically adjust (MHL) interventions using a systematic framework, such as IM Adapt, and report its use to enhance transparency in the re-use and adaptation of existing interventions which can add to the quality and comparability of evidence when evaluating interventions.

## Conclusion

In this article, we have presented the process of the systematic adaptation of an existing evidence-based MHL intervention for educational professionals in secondary education to a new cultural context, following the principles of IM Adapt. Our findings show that despite the transfer and adaptation of an existing intervention to a new context and language, only some content adaptations to LEARN were needed to address the needs and (learning) preferences of educational professionals in the Dutch education context. Therefore, existing interventions can serve as a useful basis for an adapted version, tailored to another context and population, and that this process needs to be done systematically and in collaboration with the end-user to match their needs. Such a process can lead to the transfer of the previously established program effectiveness to a new context by retaining its essential elements. Future evaluations of the effectiveness of LEARN-NL will provide more insights into the effectiveness of the intervention in this context.

## Supplementary Information

Supplementary Material 1.

## Data Availability

No datasets were generated or analysed during the current study.

## Abbreviations

MHL: Mental health literacy
IM Adapt: Intervention Mapping Adapt
