# Supplementary material for: Improving mental health literacy in secondary school educational professionals in the netherlands: adaptation of an existing intervention using Intervention Mapping Adapt
Source: BMC Public Health. 2025 Sep 2;25:3025. doi: 10.1186/s12889-025-23835-5 (PMC12403538; doi:10.1186/s12889-025-23835-5)
Supplement: Supplementary file 1 — Supplementary Material 1. [file 12889_2025_23835_MOESM1_ESM.docx]

**Appendix**

Appendix A. Planned and performed adaptations of LEARN for the Dutch context.

| **Content** |
| --- |
| Omitting module 5 on treatments and diagnoses |
| More prominent role to burn-out prevention for educational professionals (e.g., self-care strategies) (Module 7 placed earlier in intervention: now Module 2) |
| Omitting detailed theory on brain development and biological model for mental illnesses (module 4), replaced with mechanisms of ‘teenage brain’ |
| Omitted subsection on common mental illnesses in childhood |
| Omitting some learning objectives (e.g., Module 1, section 3; Module 3) |
| Reformulating some learning objectives (e.g., Module 1, section 3) |
| Omitted redundant text, reformulating text |
| Restructured Module 6 (Seeking Help Giving Support) to clarify lines of communication and mental health services inside and outside of schools |
| Added general information on treatments to Module 5 |
| Added information on social determinants for mental health to additional resources Module 1 (e.g., socioeconomic background, disability, minority groups) |
| **Delivery, design features, and cultural elements** |
| Literally translated to Dutch language, afterwards adapted to enhance comprehension in Dutch context |
| Developed digital environment (interactive pdf) |
| Cultural adaptations to fit Dutch context, specifically for education and/or mental health care system whenever needed |
| Replaced original elements with Dutch-language literature, videos, and additional information resources |
| Added Dutch subtitles to videos |
